# Increased Treatment Engagement and Adherence: Flexible Management with Prolonged-Release Buprenorphine in Treatment of Opioid Dependence

**DOI:** 10.1155/2021/6657350

**Published:** 2021-02-27

**Authors:** Bernadette Hard

**Affiliations:** Kaleidoscope Drug Project, Cardiff, UK

## Abstract

Opioid dependence (OD) is effectively treated with well-evidenced regimens including psychosocial and opioid agonist pharmacotherapy. Many do not engage with treatment services; reasons include the burden of mandatory supervision and stigma. Injectable prolonged-release buprenorphine (PRB) offers choice and flexibility in treatment. Experience reported here demonstrates the potential for PRB to enable wider engagement with treatment services. Treatment was successful in patients unable to attend daily observed therapy due to work commitments, unable to use services for fear of stigma, or having not achieved goals on previous attempts with conventional approaches. PRB therapy was clinically successful without withdrawal signs or evidence of use of other drugs. Patient-reported outcomes were positive including maintained ability to work, manageable detoxification experience, and stigma-free treatment. This work provides evidence of PRB benefit in expanding treatment engagement.

## 1. Introduction

Opioid dependence (OD) is a continuing individual and public health challenge [1] causing poor health and undesired social impact including unemployment, homelessness, family disruption, reduced economic productivity, crime, and economic burden [2, 3]. Psychosocial and pharmacotherapeutic interventions are effective [4, 5]. Approximately half of the people with problems related to opioid use do not achieve the treatment outcomes they desire and are unable to engage with the treatment system [6, 7].

Treatment with oral methadone and sublingual, or supralingual buprenorphine opioid agonists is common [8, 9]; there is an inherent risk of diversion [10]. From the start of therapy, mandatory supervised consumption is normal [9] and can make attendance at work challenging, may be a risk for associating with others involved in the provision of illicit substances [11], and is associated with discrimination [12]. These limits may explain observed suboptimal “real world” dosing, challenges in regimen adherence, and additional use, in parallel with treatment, of illicitly sourced opioids [7].

Prolonged-release buprenorphine (PRB) [13] is shown to be effective in the treatment of opioid dependence, achieving stable buprenorphine plasma levels for weekly or monthly periods after injection [14, 15], and may be a useful alternative to those unable to navigate typical oral, sublingual, or supralingual opioid agonist treatment regimes [16–18].

Cases presented describe successful initiation and continuation of treatment with PRB as an option for individuals unable to adhere to conventional treatment regimens. These insights help define situations for recommendation of this treatment option.

## 2. Case Presentation

### 2.1. Case Study 1: Treatment for person with busy work life

A 53-year-old man with a 4-year history of opioid use commenced first opioid agonist therapy with PRB, successfully ceasing smoked heroin use.

Substance use began with increasing use of codeine on prescription for a knee injury. Opioid use continued with smoked heroin use of 1 gram per day, sourced from internet suppliers with no engagement with criminal justice. The patient had a successful professional career running a demanding business. Continuing opioid use became problematic with work and relationships.

Patient was fearful of withdrawal following experience of “feeling as if dying” during planned withdrawal attempts. Attempted to cease use of opioids several times but was unsuccessful. Initial contact with standard, statutory treatment services was not successful. The only treatment option included daily supervised medication which implied extensive time travelling, not compatible with work life. The patient reported that “everything was starting to fall apart.”

On presentation at a different treatment service, the patient was anxious and reported concern about potential opioid withdrawal signs when starting any form of therapy. Urine drug screen showed possible morphine use. Treatment plan agreed with the patient beginning with sublingual buprenorphine 4 mg daily followed by a PRB 24 mg (Buvidal, Camurus AB) injection for 1 week, changing to monthly injections (left or right deltoid areas), 96 mg (Buvidal, Camurus AB).

Treatment experience was reported as satisfactory with mild opioid withdrawal symptoms (poor appetite, low energy, anxiety, and agitation) during days one to three, settling with time. Care included telephone support.

From week 1 of treatment with PRB, the patient stabilised physically and mentally with no opioid withdrawal symptoms, no cravings, and ceased use of opioid and other drug. Self-care has improved with daily exercise, yoga, and meditation. Work and relationships are manageable, with new successes in business reported. Future plans likely to include a detoxification program using an injection of PRB.

Patient-reported outcomes following 3 months of monthly PRB are favourable. The simplicity of the treatment approach is reported to enable work and family life, given the therapy being “minimally intrusive” and that “it would not be possible to carry on with normal life” if obliged to attend daily pick up for medication.

### 2.2. Case Study 2: Application in planned detoxification

A 43-year-old man, with a history of substance use from early adulthood, successfully completed opioid detoxification with an injection of 64 mg, Buvidal, prolonged-release buprenorphine.

History included more than twenty years of substance use including cocaine and injected heroin with opioid dependence. Treatment options over a period of 15 years included oral methadone therapy followed by an extended period in a therapeutic community developing a socially stable situation and an ongoing relationship. Over the subsequent 10 years, substance use increased with chaotic risk-taking behaviour, such as an attempted suicide by deliberate self-poisoning.

At presentation, following a medical assessment and urine drug screen completed, a treatment plan was agreed with the patient starting 6 mg sublingual buprenorphine, with a reducing course of diazepam, and a 4-month residential rehabilitation course.

With further discussion, it was planned to attempt a single administration of monthly prolonged-release buprenorphine selected to assist with detoxification during rehabilitation. On agreement, treatment was switched from daily 6 mg sublingual buprenorphine to monthly 64 mg PRB (Buvidal, Camurus AB), administered by subcutaneous injection in the abdomen.

During treatment, the patient reported no emerging opioid withdrawal symptoms and no unwanted adverse effects. The reported treatment experience was positive, benefits identified spontaneously during reflection included: the absence of stigma associated with previous methadone observed therapy, and the ability to maintain the normal daily routine while on treatment. Since treatment, the patient is hopeful and optimistic with stable mood, employed, and in a relationship, continuing step-down dialectical behaviour therapy as outpatient, remaining abstinent from use of illicit substances.

### 2.3. Case Study 3: Positive outcomes with treatment adherence

A 34-year-old man with a history of drug use since adolescence, completed 12 consecutive months of treatment with monthly injected prolonged-release buprenorphine, enabling him to improve his quality of life and to desist from crime.

Substance use history reported illicit use of opioids, including injected heroin, for greater than 20 years. History of contact with criminal justice system begun with a period of detention aged 15 and subsequent extended periods of incarceration as an adult.

Treatment for substance use disorder including opioid dependence was intermittent and unsatisfactory for the patient, with entry to prison often disrupting therapy. Treatment history included initially methadone use and more recently fast dissolving oral buprenorphine.

During periods of treatment on methadone, use of other opioids was common; in general, treatment engagement was variable often due to difficulty in attending medication collection appointments. Similarly, prior to PRB, patient attempted treatment with fast dissolving oral buprenorphine, with ongoing use of injected heroin. Life was increasingly chaotic during this period, and the patient was often unable to attend appointments to collect medication. Following a crisis, culminating in heroin overdose precipitating a cardiac arrest, the patient decided to begin therapy with PRB.

At treatment initiation, patient was motivated to attempt prolonged-release buprenorphine to simplify therapy. Patient agreed to begin PRB therapy starting with a weekly treatment of 16 mg (Buvidal, Camurus), later switching to a monthly injection (64 mg, Buvidal).

Patient continued PRB treatment for 12 months, with no missed doses. Urine drug screening performed eight times resulted negative for illicit drug use. During the PRB treatment period, no adverse events were reported. Patient-reported outcomes included stable relationships and housing, ability to engage in hobbies, and ceased criminal activities. Treatment experience was “very positive.”

## 3. Discussion

The evidence from the three case studies describes benefits of the features and flexibility of prolonged-release buprenorphine therapy for opioid dependence for patients unable to engage with conventional treatment options.

The three cases show, from starting therapy at induction with weekly injections to continuing with monthly dosing, that PRB is well tolerated and not associated with signs of withdrawal. Patient-reported outcomes were positive: the ability to continue work, the manageable experience of detoxification, and treatment without stigma are highlighted. These are important clinical scenarios managed successfully, which are often challenging to address, and are examples of situations where other choices of oral pharmacotherapy were not optimal.

Oral buprenorphine or methadone treatment for opioid dependence is effective with limitations. A significant proportion of patients is unable to engage with treatment and does not achieve the results desired. It is hard to maintain activities of normal living while engaged with observed therapy and regular collection of medication. The process of therapy is often associated with stigma, risks, poor adherence, drop-outs from treatment, and relapse. PRB with its flexible weekly and monthly options makes it easier for patients to engage with therapy. Understanding the impact of such new choices in the system widens treatment engagement and is key to expand treatment to all who want it.
